# “Brain-First” vs. “Body-First” PD: Definitions and Implications in Everyday Clinical Practice: A Systematic Review

**DOI:** 10.3390/medicina62061116

**Published:** 2026-06-08

**Authors:** Ioannis Pilateris, Sevasti Bostanjopoulou

**Affiliations:** 1 Department of Neurology, Aristotle University of Thessaloniki, 54124 Thessaloniki, Greece; 2 “KAT” Attica General Hospital, 14561 Athens, Greece; 3 Department of Neurology, Aristotle University of Thessaloniki, George Papanikolaou Hospital, 57010 Thessaloniki, Greece; bostkamb@otenet.gr

**Keywords:** Parkinson’s disease, brain-first PD, body-first PD, pathophysiology, disease progression, biomarkers, microbiome, prognosis

## Abstract

*(1) Background and Objectives:* Parkinson’s disease’s (PD) underlying pathophysiology still remains incompletely understood, with Braak’s hypothesis of ASyn pathology propagation being the most widely accepted. Recently, a novel model has been introduced, proposing two distinct ASyn propagation pathways: a bottom-up trajectory termed Body-first PD, and a central nervous system (CNS)-initiated pathway termed Brain-first PD. This distinction introduces new perspectives in the PD literature landscape regarding diagnosis, prognostic factors and patient management. This study set out to systematically synthesize the current literature comparing Brain-first and Body-first PD, with a focus on clinical characteristics and disease progression, diagnostic biomarkers, and management approaches. *(2) Materials and Methods:* A systematic literature search was conducted in March 2025 using PubMed, Cochrane Library, DOAJ and Google Scholar. Human observational, diagnostic, and interventional studies published between 2019 and March 2025, including patients with de novo or early PD, were eligible. Pre-motor REM sleep behavioral disorder (RBD) was used as the primary differentiation criterion. Risk of bias was evaluated using the Joanna Briggs Institute (JBI) critical appraisal checklists. Results were synthesized using a narrative approach. *(3) Results:* Sixteen studies comprising 2107 PD patients met the inclusion criteria. Body-first PD was associated with a higher non-motor symptom (NMS) burden, faster disease progression, and a higher prevalence of cognitive impairment. Additionally, Body-first PD patients exhibited more widespread and symmetrical neurodegeneration, along with electrophysiological and metabolic differences. Distinct biomarker and microbiome profiles were also observed between subtypes. No eligible studies addressing management approaches were identified. *(4) Conclusions:* In conclusion, the available evidence suggests that Brain-first and Body-first PD may represent two distinct pathophysiological entities, a proposal with great significance for the diagnosis, prognosis and management of PD patients. However, the predominantly cross-sectional nature of the current literature limits causal inference. Future longitudinal and interventional studies are required to clarify the potential clinical implications of this subtype classification theory.

## 1. Introduction

Parkinson’s disease (PD) was first documented in 1817 by James Parkinson as Shaking Palsy (“Paralysis agitans”). Despite over two centuries of investigation, its pathophysiology remains incompletely understood and is a subject of extensive research to this day. The central feature of PD pathophysiology seems to be the presence of intraneuronal protein inclusions containing aggregated alpha-Synuclein (ASyn), commonly referred to as Lewy pathology (LP). The accumulation of these inclusions within dopaminergic neurons is believed to promote neurodegeneration through multiple mechanisms, including neuroinflammation, oxidative stress and excitotoxicity, among others [1,2]. Emerging evidence also suggests that metabolic disturbances, including glycemic variability, may contribute to neurodegeneration and influence disease vulnerability [3].

A major conceptual breakthrough occurred in 2003, when a staging theory was proposed for sporadic PD, based on the observation that ASyn pathology spreads throughout the central nervous system (CNS) in a non-random, predictable pattern [4]. According to the model, LP initially appears in the dorsal motor nucleus of the vagus and glossopharyngeal nerves, as well as the anterior olfactory nucleus (Stage 1). It thereafter ascends the CNS, targeting specific vulnerable regions, starting from the brain stem.

**Figure 1:**
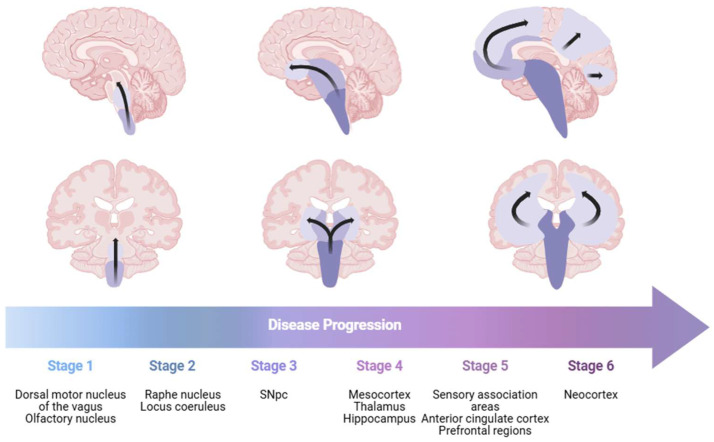
Schematic representation of Parkinson’s disease progression according to Braak’s staging hypothesis. The figure illustrates the sequential involvement of regions from the lower brainstem (Stages 1–2), through the midbrain including the substantia nigra pars compacta (SNpc) (Stage 3), limbic and mesocortical regions (Stages 4–5), and finally widespread neocortical areas (Stage 6). Arrows indicate the proposed trajectory of LP propagation, while color density reflects the relative burden of LP.

At Stage 2, pathology extends to the caudal raphe nuclei, gigantocellular reticular nucleus, and coeruleus–subcoeruleus complex. Subsequent involvement of the substantia nigra pars compacta (SNpc) characterizes Stage 3 and coincides with the first motor symptom manifestations of PD. Lesions on the basal prosencephalon and the mesocortex through the anterior olfactory nucleus are first observed at Stage 4, with additional involvement of other midbrain nuclei, including the paranigral and the pigmented parabrachial nucleus. At this stage, LP is also detected on the hippocampus and anteromedial temporal mesocortex. Neocortical involvement begins in Stage 5, progressing from the temporal mesocortex to sensory association areas, the granular and agranular insular fields, the anterior cingulate cortex, and prefrontal regions. Finally, in Stage 6, LP becomes widespread throughout almost the entire neocortex, while the pre-motor areas, primary motor and sensory cortex are relatively spared (Figure 1).

This hypothesis is further supported by the presence of pre-motor symptoms associated with early disease stages (such as constipation or olfactory disorders) [5,6], as well as the relatively well-established clinical progression of the disease [7]. However, other studies reported contradictory findings, i.e., a proportion of patients not conforming to Braak’s staging scheme or not harboring Lewy pathology in the dorsal motor nucleus of the vagus on postmortem studies [8,9].

Recent evidence suggests that in a subset of PD patients, ASyn pathology does not follow the same retrograde vagal propagation pattern described in Braak’s hypothesis [10]. It instead appears to originate within the CNS, with early nigrostriatal involvement preceding significant peripheral nervous system (PNS) damage. Clinically, these patients often do not manifest REM sleep behavioral disorder (RBD), a common prodromal feature of PD, until after the onset of motor symptoms. This observation is supported by both imaging and histopathological evidence, demonstrating sparing of the dorsal motor nucleus of the vagus in patients with marked SN involvement [11,12].

Consequently, the “a-Synuclein Origin Site and Connectome” (SOC) model was proposed [13]. It underlines two key factors that heavily impact Asyn propagation: the initial site of Asyn pathology and the most dominant connections facilitating its spread. Within this context, two distinct PD subtypes have emerged, known as “Brain-first” (or CNS-first) and “Body-first” or “Gut-first” (or PNS-first) PD. The present systematic review aims to summarize the existing literature comparing these two subtypes, with the objective of evaluating any clinical significance in considering them distinct entities.

Emphasis is placed on differences in diagnostic biomarkers, disease progression and prognosis, as well as implications for patient management.

## 2. Materials and Methods

### 2.1. Eligibility Criteria

An extensive literature search was performed in March 2025. Studies published between January 2019 and March 2025 were considered eligible for inclusion, as 2019 corresponds to the emergence of the ”Brain-first” vs. “Body-first” PD theory. Only studies published in English were considered, since translation resources were unavailable.

We included human observational, interventional, and diagnostic studies involving de novo or early idiopathic PD patients, defined as a mean disease duration less than 6 years, with an age range of 40–80 years. Studies were required to compare Brain-first and Body-first PD subtypes. As no universally accepted definition of the two subtypes currently exists, subtype classification was based on the most widely used criteria: the presence of pre-motor RBD (PDRBDpre) defining the Body-first subtype, and the absence of pre-motor RBD (PDRBDpost or PDRBD-) defining the Brain-first subtype. We also considered evidence of Cardiac Sympathetic Denervation (CSD) assessed by ^123^I-metaiodobenzylguanidine (MIBG) scintigraphy as a secondary classification criterion when available, enhancing differentiation accuracy.

The following were excluded: animal studies, studies on atypical or familiar Parkinsonism and other neurodegenerative diseases, case reports, case series, narrative or systematic reviews, and non-relevant gray literature. This review was conducted in accordance with the PRISMA 2020 checklist guidelines [14].

### 2.2. Search Strategy

Three primary databases (PubMed, Cochrane Library, and DOAJ) and one secondary database (Google Scholar) were searched by one reviewer (I.P.). Due to the limited number of eligible studies identified in the primary databases, Google Scholar was included to ensure comprehensive coverage. All search results were independently double-checked by a second reviewer (S.M.).

The search strategy was initially implemented and trialed in PubMed, with minimal adaptations for compatibility with the remaining databases. For Google Scholar searches, only the first 1000 retrieved records were screened, as Google Scholar only provides access to the first 1000 search results, presented according to the platform’s relevance-based ranking algorithm. Therefore, retrieving articles beyond this threshold was not feasible. Gray literature was searched selectively and only included if relevant for the objective of the review. All retrieved articles were imported into Zotero [15] reference management software (v. 7.0.32) for organization, duplicate removal, and citation management. The full search strategy, including keywords and Boolean operators, is provided in Supplementary Table S1.

### 2.3. Data Extraction

Data was selected and extracted independently by one reviewer (I.P.) and subsequently verified for accuracy and transparency by a second reviewer (S.B.). Extracted data included study characteristics (author, country, year of publication, study setting), population characteristics (sample size, age, gender distribution, PD diagnostic criteria, disease duration, relevant demographic differences), intervention or exposure, comparators, outcomes assessed and key and secondary results.

Titles and abstracts were screened using Rayyan [16], an online systematic review platform that facilitates blinded screening and efficient conflict resolution. No automation tools were used for article exclusion. In cases where data were unavailable, corresponding authors were not contacted for additional information or full article access.

Outcomes were categorized into four predefined domains: diagnostic approach and biomarkers, microbiome differences, prognosis and disease progression (motor and non-motor manifestations), and management approach. Three studies had incomplete population data—PD diagnostic criteria (*n* = 2), age distribution (*n* = 1), sex distribution (*n* = 2), precise disease duration (*n* = 1)—but were retained for the following reasons: reliance on the Parkinson’s Progression Markers Initiative (PPMI) database [17] (*n* = 2), and the limited relevance of sex distribution given the relatively small sample size (*n* = 1).

### 2.4. Quality Assessment

Methodological quality and risk of bias for each included study were assessed using the Joanna Briggs Institute (JBI) critical appraisal checklists [18], according to each study design. This approach evaluates internal validity and identifies potential sources of bias within individual studies. Studies were considered adequate if they achieved a score of ≥7 for longitudinal studies, or ≥5 for cross-sectional studies.

### 2.5. Data Synthesis

Included studies were tabulated according to the study design, subtype definition method, and outcomes assessed. Studies were thereafter grouped by outcome domain as the preferred method, for a more robust comparison of the results. Primary outcomes measured included the following: clinical characteristics and disease progression, imaging evidence and differences, biomarker differences, microbiome alterations and management approach. Due to the limited number of eligible studies and substantial heterogeneity, synthesis was performed using a narrative approach, incorporating all relevant and widely available data.

### 2.6. Reporting Bias and Certainty of Evidence Assessment

A formal assessment of reporting bias was not conducted; however, it will be considered qualitatively and addressed in the Discussion as part of the study limitations. Certainty of evidence was evaluated using the GRADE framework [19], supported by the interactive Summary of Findings (iSoF) online tool. Five GRADE domains were assessed: risk of bias, inconsistency, imprecision, indirectness, and publication bias.

## 3. Results

### 3.1. Overview

**Figure 2:**
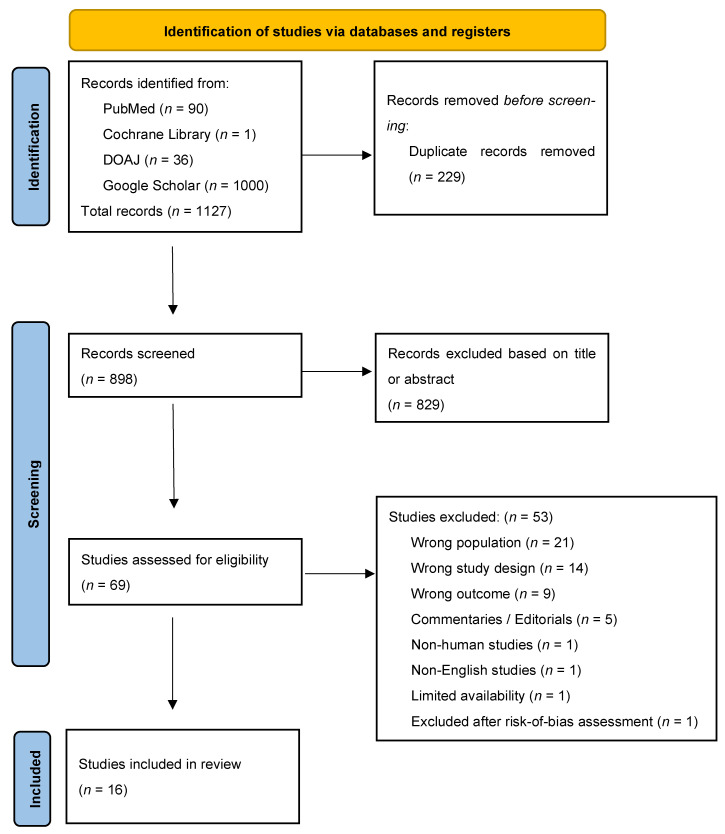
Flowchart depicting the assessment protocol of eligible studies according to PRISMA guidelines.

A total of 1127 records were identified through the initial database search. Following duplicate removal, 898 remained for screening. Title and abstract screening yielded 69 studies meeting the inclusion criteria, which underwent full-text evaluation. After full-text assessment and risk-of-bias appraisal, 16 studies were deemed eligible and were therefore included in the final synthesis [20,21,22,23,24,25,26,27,28,29,30,31,32,33,34,35]. The complete study selection process is illustrated in the PRISMA flow diagram [Figure 2].

The 16 included studies comprised a total of 2107 PD patients, of whom 713 were classified as Body-first PD and 963 as Brain-first PD. As previously stated, the presence of pre-motor RBD is widely considered the principal criterion for subtype dichotomization, while CSD assessed through MIBG scintigraphy has been proposed as an important supportive feature. Therefore, in studies where explicit subtype nomenclature was not used, subtype classification was inferred primarily based on the presence of pre-motor RBD, with CSD findings on MIBG scintigraphy considered a secondary supportive criterion, when available. One study included 419 PD patients from the PPMI database without subtype classification, as a comparative group against individuals with idiopathic RBD (iRBD), representing a mixed PD cohort (Brain-first and Body-first) compared to a putative pure Body-first PD group [26]. Additionally, one study included a subgroup of 12 “Undetermined” PD patients, defined by discordance between RBD status and MIBG scintigraphy results [31]. Three studies included a total of 121 iRBD individuals. Seven studies incorporated 564 healthy controls (HCs).

Among the included studies, three (*n* = 3) were cohort and thirteen (*n* = 13) were cross-sectional studies. One study from the gray literature (non-peer-reviewed preprint) was included following careful consideration of its methodological relevance and contribution to the review objectives [35]. There was considerable variability in subtype distribution: four studies included a higher proportion of Body-first PD patients, eleven studies included more Brain-first PD patients, while one study matched the two groups for sample size and demographic factors. Our review included studies from China (*n* = 5), Italy (*n* = 4), South Korea (*n* = 3), Germany (*n* = 3) and the USA (*n* = 1). Three studies utilized data from the PPMI database, including one study that provided two datasets for comparability. Considering the diagnostic criteria for PD, six studies applied the United Kingdom (UK) PD Society Brain Bank criteria [36], six implemented the Movement Disorder Society (MDS) diagnostic criteria [37], one study adopted both criteria depending on enrollment date, two studies did not report specific criteria (both used the PPMI database), while one study stated the diagnosis was made by a Movement Disorders Specialist. Subtype classification methods varied across studies. Ten studies relied on RBD screening questionnaires, including the RBD Screening Questionnaire (RBDSQ) [38] (*n* = 9) and the RBD single-question screen (RBD1Q) [39] (*n* = 1), with some incorporating additional questions to improve specificity and symptom onset accuracy. Only one study employed the gold-standard polysomnography (PSG) [40,41], while three studies used the RBDSQ in combination with PSG for establishment of the RBD diagnosis. In two studies, subtype classification was based on both the RBD status and CSD. One of these adopted the RBD1Q screening tool with supplementary symptom onset-related questions alongside CSD in MIBG scintigraphy, while the other combined RBDSQ with MIBG scintigraphy.

Finally, although all included studies focused on patients with a mean disease duration of less than six years, we observed a significant discrepancy in disease duration. Specifically, ten studies enrolled “de novo” PD patients (mean disease duration under two years), whereas six included patients with early-stage PD (mean disease duration between two and six years).

Unfortunately, no longitudinal studies assessing management approaches met the inclusion criteria. Consequently, the included studies were categorized into four primary outcome domains: Clinical characteristics and disease progression (*n* = 4), Imaging evidence and differences (*n* = 8), Biomarker differences (*n* = 2), and Microbiome differences (*n* = 2). A comprehensive summary of extracted study characteristics is provided in Table 1.

### 3.2. Risk-of-Bias Assessment

Of the 17 studies initially assessed for methodological quality with the JBI critical appraisal tools, one failed to meet the predefined cutoff value (4/8) and was therefore excluded from the review [42]. The remaining 16 studies satisfied the quality criteria and were included in the final synthesis. A summary of the appraisal results for the included studies is presented in Supplementary Table S2, with detailed individual assessments provided in Supplementary Figure S1.

### 3.3. Clinical Characteristics/Disease Progression

As for demographic characteristics, the Body-first PD group exhibited a higher proportion of male patients, a significantly longer disease duration at presentation and at last follow-up, a younger age at enrollment and at onset, and a higher mean Levodopa Equivalent Dose (LED) in one study each when compared to the Brain-first PD group. Conversely, four studies reported an older age at enrollment in the Body-first PD group, while a lower LED and shorter disease duration at last follow-up were reported in one study each. Nine studies found no statistically significant differences in disease duration, age, or sex distribution, and four studies found no difference in LED between groups.

Regarding motor symptoms, Body-first PD patients showed significantly lower United Parkinson’s Disease Rating Scale part III (UPDRS-III) asymmetry indices (AIs) in two studies and a lower UPDRS-III score at presentation in one study. In contrast, they demonstrated higher UPDRS-I scores and worse Parkinson Disease Questionnaire 39 (PDQ-39) performances in two studies, as well as higher UPDRS-II scores and higher gait and postural scores in the MDS-UPDRS III in one study each. Seven studies reported no statistically significant differences between the two groups in Hoehn–Yahr (H&Y) Stage or UPDRS-III scores at presentation. Furthermore, no differences in PDQ-39 scores, UPDRS total scores, UPDRS-II scores, or UPDRS-IV scores were reported in one study each.

Body-first PD patients presented a significantly faster worsening in UPDRS-III scores per month when compared to Brain-first PD patients in one study, whereas in another study, there was no difference in PDQ-39 score progression rates.

One study reported no associations between RBD and dyskinesias or wearing off in either group. Another study found a positive correlation between RBD-HK and MDS-UPDRS scores and PDQ-39 scores.

With regard to non-motor symptoms, Body-first PD patients had higher Non-Motor Symptoms Scale (NMSS) scores at baseline in four studies, as well as higher scores in the gastrointestinal domain of the NMSS on two studies, and in the sleep/fatigue domain of the NMSS on one study. Moreover, the group presented higher Scales for Outcomes in Parkinson’s Disease-Autonomic Dysfunction (SCOPA-AUT) scores at presentation in two studies and during all intervals of follow-up in one study, and higher cardiovascular and gastrointestinal SCOPA-AUT scores and a higher prevalence of autonomic dysfunction (as defined by SCOPA score > 12) at 5 years of follow-up in one study. They also demonstrated a higher (but not statistically significant) prevalence of orthostatic hypotension and lower olfaction scores in one study each. Furthermore, Body-first PD patients exhibited significantly lower voiding volumes and first desire volumes (FDVs), a higher (albeit not statistically significant) prevalence of detrusor overactivity, significantly higher urological questionnaire scores [Overactive Bladder Symptom Score (OABSS), International Prostate Symptom Score (IPSS), and IPSS-storage (IPSS-s)], higher maximum voided volumes and a higher prevalence of overactive bladder (OAB), according to OABSS, each in one study.

Additionally, Body-first PD patients had higher Hamilton Depression Rating Scale (HDRS) scores in three studies, as well as a consistently higher depression prevalence, higher mean State-Trait Anxiety Inventory (STAI) scores, significantly higher Hamilton Anxiety Scale (HAMA) scores, a significantly higher prevalence of hallucinations with faster severity progression after four years, a higher prevalence of apathy (statistically significant after two years), and higher Questionnaire for Impulsive-Compulsive Disorders (ICD) in Parkinson (QUIP) scores in one study each.

In contrast, three studies found no statistically significant differences in Beck Depression Inventory (BDI) scores, two studies found no differences in Epworth Sleepiness Scale (ESS) scores, while no differences in total SCOPA-AUT scores and in the urinary, thermoregulatory, pupillary, and sexuality components of SCOPA-AUT, in NMSS, ESS or BDI progression rates, in the frequency of orthostatic hypotension, in ROME-III constipation module scores and in olfaction scores were reported in one study each. Urodynamic parameters [maximal flow rate (Qmax), detrusor pressure at Qmax (PdetQmax), post-void residual volume (PVR)], the prevalence of detrusor underactivity, compliance, or bladder outlet obstruction, as well as voiding diary parameters and the urinary parameters of NMSS questionnaires, were also comparable between groups in one study.

One study reported a non-significant positive association between hallucinations and RBD, more pronounced and significant in Body-first PD. Another reported a positive correlation between RBD-HK and HDRS as well as Activities of Daily Living (ADL) scores. One study also found a positive correlation between a delayed MIBG-scintigraphy H/M ratio and voiding volume, a negative correlation with OABSS (total and in-storage dysfunction parameters), IPSS and IPSS-s scores, and an independent association with the voiding volume and FDV.

One study reported significant hazard ratios (HRs) for the presence of probable RBD (Body-first subtype): depression, STAI score, QUIP score, and SCOPA score.

Considering cognitive parameters, Body-first PD patients demonstrated a higher frequency of cognitive impairment in three studies [two addressing Mild Cognitive Impairment (MCI) only, one addressing both MCI and dementia]. One study reported that while the cognitive impairment prevalence was comparable between groups in the first two years, after the two-year time period, it was significantly higher in the Body-first PD group. A faster decline in Montreal Cognitive Assessment (MoCA) and Symbol Digit Modality (SDM) scores in Body-first PD patients was also reported in one study.

Conversely, five studies found no significant differences in Mini-Mental State Examination (MMSE) scores or MoCA scores. Other cognitive test scores, including Clinical Dementia Rating (CDR), Frontal Assessment Battery (FAB), SDM, Hopkins Verbal Learning Test (HVLT), Letter Number Sequencing (LNS) test, Benton Judgment of Line Orientation (BJLO) test, and Semantic Fluency Test (SFT), were also similar at presentation in one study each. Furthermore, HVLT, LNS test, BJLO test, SFT scores, and MMSE progression rates were also comparable throughout follow-up, each in one study.

One study reported a positive association between RBD and MCI, stronger and significant in Body-first PD, while no association was found with memory, attention, visuo-spatial or executive domains, even though Body-first PD patients had overall higher odds of impairment in attention and memory.

Overall, certainty of evidence for clinical characteristics was rated low, downgraded due to observational study designs, inconsistency of results, and imprecision (small sample size).

### 3.4. Imaging Evidence/Differences

Body-first PD patients exhibited significantly more remaining radiopaque markers in colonic transit examinations in two studies, as well as significantly lower late and early image MIBG H/M ratios and washout rates, significantly lower colonic 11C-donepezil Standardized Uptake Values (SUVs), and significantly enlarged colon volumes in one study each.

Meanwhile, no significant differences in intracranial volume (ICV) or gray matter volume (GMV) were observed in one study.

One study found no correlations between RBD symptom duration or RBDSQ scores and MIBG, donepezil, colon volume, or transit data.

One study compared 18F-FDG PET imaging findings among Body-first PD, Brain-first PD patients, and HCs. The Brain-first PD group showed decreased uptake in the left middle occipital, left superior occipital and left inferior parietal gyri, and increased uptake in the precentral gyrus, right paracentral lobule, and cerebellum, when compared with HCs. Meanwhile, the Body-first PD group showed decreased uptake in the left middle frontal, left middle temporal, and left middle occipital gyri, with an increase in the paracentral lobule, putamen, thalamus, and the cerebellum, when compared to HCs. Finally, Body-first PD patients showed reduced uptake in the left inferior parietal, left middle temporal, and bilateral middle occipital gyri, with no significant increases, when compared with the Brain-first PD group.

When examining dopaminergic dysfunction and asymmetry, Body-first PD patients exhibited more symmetric putaminal involvement in three studies (while caudate nucleus involvement was also more symmetric in one study), as well as a decreased (though not statistically significant) Neuromelanin MRI mean Locus Coeruleus (LC)/pons ratio, lower mean caudate and putamen-specific binding ratios (SBRs) in FDOPA PET, a higher striatum/pons binding ratio and significantly lower striatum and pons volumes of interest (VOIs) in FP-CIT PET, and significantly lower FP-CIT PET standardized uptake ratios (SURs) in the least affected hemisphere (LAH) putamen, caudate and ventral striatum [while SURs in the most affected hemispheres (MAHs) were similar] in one study each. One study comparing both Body-first and Brain-first PD patients to HCs reported greater alterations in LC and pons functional connectivity (FC) on resting-state functional MRI (rs-fMRI) in the Body-first PD group. This study also reported a reduced FC between the left LC and all five resting-state networks (RSNs) examined, and between the right LC and default mode network (DMN), salience (SAL), and sensorimotor network (SMN) when comparing the Body-first PD with the Brain-first PD group. On the other hand, two studies reported no significant differences in putamen SBRs or in striatal AIs between groups, while no significant differences in the MAG gray matter volume (MAHGMV), LAH gray matter volume (LAHGMV), or gray matter volume asymmetry index (GMV AI) were also reported in one study each.

One study found that in Body-first PD, FC between the left LC and DMN and visual network (VIS) was positively associated with MoCA scores, while FC between the right LC and SAL was negatively correlated with total RBDQ-HK scores and RBD (Q6–Q12) scores. In Brain-first PD, FC between the left LC and DMN correlated negatively with HDRS scores. No significant correlations were found between pons FC with all five RSNs and any clinical assessments in all groups. One study reported a significant positive association between PDRBDpre and mean caudate SBR and putamen AI, and another study found no correlations between putamen AI and total RBDSQ scores.

As previously mentioned, one study compared iRBD patients with a cohort of mixed Body-first/Brain-first PD patients. While median SBRs of iRBD patients were less pathological at baseline and at the one-year follow-up, the values converged with the PD cohort at the two- to five-year intervals. Median putaminal AI of iRBD patients was consistently lower at all time points. Furthermore, based on the most recent pathological SPECT scan of iRBD patients, SBRs did not differ between groups, but the uptake was again significantly more symmetric in the iRBD group. It is also worthwhile noting that the UPDRS difference scores indicated markedly more symmetric parkinsonian symptoms in iRBD-converters than the whole PD cohort.

One study further categorized PDRBDpre patients according to the duration of pre-motor RBD (PDRBDpre ≥ 10 yrs and PDRBDpre < 10 yrs). Both groups had lower pontine FP-CIT uptakes compared to the PDRBD- group, but putaminal FP-CIT binding reduction was observed only in the PDRBDpre < 10 yrs group. The three groups exhibited comparable values in caudate uptake. The PDRBDpre < 10 yrs group also showed a prominently symmetric putamen and caudate nucleus denervation pattern when compared to PDRBD–, while PDRBDpre ≥ 10 yrs showed relative symmetry only in the putamen. When examining the aspect of asymmetry, arithmetically higher (with no statistical significance) asymmetries were found in the PDRBDpre ≥ 10 yrs than the PDRBDpre < 10 yrs group.

Regarding neurophysiological studies, one study reported an abnormal α frequency network with lower mean network connectivity (mNC) values in Body-first PD patients, when compared with HCs. The regions of interest (ROIs) when comparing to HCs comprised of the prefrontal, orbitofrontal, sensorimotor, and temporal cortexes, while ROIs of difference between the Body-first and Brain-first PD subgroups included the prefrontal and temporal lobes, as well as the anterior cingulum. No significant differences in the AI of this network were found between groups. Another network in the β band was also found that differed significantly between the PD and HC groups. Its ROIs included the sensorimotor and limbic regions, and to a lesser extent, the parietal lobes. Both PD subgroups showed higher mNC in this network when compared to HCs, but when compared to each other, mNC values were similar. Body-first PD patients presented significantly lower AI in this altered β network.

Another study found that after EEG spectral analysis in the resting state, Body-first PD patients showed significantly lower values of Global Power Spectral Density (G-PSD), as well as lower β values in multiple frontal, temporal, and parietal regions. This study also observed a significant trend of β-band reduction (PD-RBD- > PD-RBDpost > PD-RBDpre) in both global and site-specific power in multiple frontal, temporal, parietal and occipital regions. Conversely, no differences between groups were found in the θ and low-γ frequency bands or when comparing δ, θ and α absolute powers were found in individual studies.

One study reported a significant positive correlation between the abnormal α network mNC and MoCA scores in Body-first, and, to a lesser extent, Brain-first PD patients, and a negative relationship between the mNC and MDS-UPDRS-III gait/postural subscores in Body-first PD patients only. A positive correlation was also reported between the β network mNC and MDS-UPDRS-III bradykinesia subscores in both groups.

Another study found a significant positive association between low G-PSD beta band values and PD-RBDpre, whereas no significant association was found in other frequency bands.

Certainty of evidence was considered low, downgraded for an observational study design, inconsistency of results, variability across imaging modalities, imprecision, and indirectness of outcomes.

### 3.5. Biomarker Differences

One study reported significantly elevated plasma GFAP and NfL levels in both PD subtypes, with Body-first PD patients showing significantly elevated levels compared to the Brain-first PD group. Meanwhile, no significant differences were identified in plasma Tau and pTau-181 levels.

In the Brain-first PD group, plasma GFAP levels demonstrated positive correlations with 18F-FDG uptake in bilateral substantia nigra and right cerebellum, and negative correlations with uptake in the left inferior and superior parietal gyri. Plasma NfL levels correlated positively with 18F-FDG uptake in the bilateral cerebellum, and negatively with uptake in bilateral superior frontal and anterior cingulate gyri, cuneus and thalamus. No correlations were observed in the Body-first PD group.

Another study reported a significantly reduced right and left vagus nerve (VN) cross-sectional area (CSA) in Body-first PD patients when compared to Brain-first PD patients.

Right VN CSA showed a moderate negative correlation with the Body-first PD subtype and RBDSQ, and a weak negative correlation with the H-Y stage, UPDRS total score, UPDRS-III, NMSQ, PDQ-39, SCOPA-AUT total score and its cardiovascular and gastrointestinal components, as well as a weak positive correlation with the PDSS score. Meanwhile, the left VN CSA showed similar moderate negative correlations with the Body-first PD subtype and RBDSQ, and weak negative correlations with the H-Y stage, PDQ-39, SCOPA-AUT total score and its gastrointestinal component.

One study reported altered bile acid composition profiles across the PD subtypes. Regarding the PD group as a whole, significant differences were found for glycochenodeoxycholic acid (GCDCA), glycocholic acid (GCA) and glycodeoxycholic acid (GDCA) in comparison with HCs. When comparing Body-first PD patients to HCs, significant differences were observed in GCDCA, GDCA, and taurochenodeoxycholic acid (TCDCA) levels. Finally, only GDCA levels were significantly different between the Brain-first PD group and HCs. Nevertheless, no significant differences were found between the two PD subtypes.

One study identified 411 upregulated and 214 downregulated microbiome gene biomarkers in the Body-first PD group when compared to Brain-first PD. Increased biomarkers were mostly associated with structural proteins of Curli fimbriae and extracellular polysaccharides, secretion and nucleation pathways, and ASyn aggregation through Toll-like receptor-activated inflammatory responses, i.e., lipopolysaccharide biosynthesis, flagella assembly, and bacterial invasion of epithelial cells. On the other hand, decreased biomarkers were involved in spermidine and short-chain fatty acid (SCFA) biosynthesis pathways, i.e., acetate, propionate, and butyrate biosynthesis.

Certainty of evidence for biomarker outcomes was rated low, as it was downgraded due to an observational study design, indirectness (surrogate outcomes), imprecision, and inconsistency of results between groups.

### 3.6. Microbiome

On the subject of the microbiome, one study found significantly different β-diversity profiles in Body-first PD patients, who also presented a less similar microbiome composition compared to HCs than the Brain-first PD group. Specifically, they presented a lower abundance of *Firmicutes* and *Bacteroidetes*, and a higher abundance of *Actinobacteria*, *Proteobacteria*, and *Verrucomicrobia* when compared to both Brain-first PD and HCs at the phylum level. Conversely, another study reported no significant differences in α- or β-diversity indexes between the two groups.

At the genus level, Body-first PD patients showed a lower abundance of Blautia, Ruminococcus 1, Dorea and Eubacterium ventriosum group, and a higher abundance of Campylobacter and Veillonella when compared to Brain-first PD patients in one study each.

One study also identified significant differences between the two groups in the genera Acidaminococcus, Alloscardovia, Fournierella and Phocea.

At the species level, one study identified 43 increased and 24 decreased species in the microbiome of Body-first PD patients, in comparison to the Brain-first PD group. Commonly increased species included *Escherichia coli* group, *Escherichia fergusonii*, *Enterobacter hormaechei*, and *Akkermansia muciniphila*, while decreased species included *Roseburia faecis*, *Roseburia intestinalis*, *Agathobacter rectalis*, and *Faecalibacterium duncaniae*.

On this topic, the certainty of evidence was considered low, downgraded for study limitations (variability in analysis methods and observational study design), imprecision (small sample sizes), inconsistency between studies, and indirectness (surrogate outcomes).

### 3.7. Secondary Outcomes

Apart from the between-group comparison, some secondary data were also collected.

Several studies included data concerning iRBD patients, representing a potential prodromal Body-first PD phenotype. One study reported lower NMSS total and gastrointestinal (GI) scores when comparing iRBD patients to the total PD group. Another study reported a reduced FC of the left LC with all five major RSNs, while the right LC showed a reduced FC with the DMN and executive control nucleus (ECN) in iRBD patients versus HCs. When comparing iRBD patients with the Brain-first PD group, the former demonstrated reductions in the FC between the left LC and DMN, ECN, and VIS, as well as in the FC between the right LC and SMN. Another study reported significantly less pathological putamen SBRs in iRBD patients when compared with the Brain-first PD group.

Finally, one study evaluated the diagnostic capacity of VN CSA. For PD diagnosis, the area under the curve (AUC) was 0.840 for right VN CSA and 0.778 for left VN CSA. Cut-off values for the right VN CSA were 2.35 mm^2^ (0.87 sensitivity), and for the left 1.75 mm^2^ (0.83 sensitivity), as derived from the ROC curve. For distinction between subtypes, AUC was 0.776 for right VN CSA and 0.723 for left VN CSA. Cut-off values were 1.82 mm^2^ (0.74 sensitivity) and 1.45 mm^2^ (0.70 sensitivity), respectively.

### 3.8. Certainty of Evidence

After careful evaluation, overall certainty of evidence across all domains was rated low, primarily due to the small number of available studies, observational study designs, and inconsistency of results. A detailed summary of certainty assessments following the GRADE-iSoF approach is provided in Supplementary Table S3.

## 4. Discussion

Even though PD has been the subject of extensive research for many years, the Body-first versus Brain-first PD hypothesis has been in the spotlight only recently. Despite its relative novelty, a growing amount of literature has rapidly accumulated, reflecting a renewed interest in the potential clinical implications of this proposed dichotomy. The present systematic review aimed to summarize available data concerning those implications across clinical, imaging, biomarker, and microbiome domains.

Although evidence is predominantly based on cross-sectional data—reflecting the limited time since the hypothesis’ inception—it is possible to at least make some speculations concerning the two subtypes. Collectively, the findings suggest that Body-first PD may clinically represent a more malignant subtype, characterized by a lower motor symptom but higher non-motor symptom burden at early disease stages, followed by faster disease progression across both domains, and a higher incidence of cognitive impairment, albeit with debatable findings regarding specific cognitive parameters. Across multiple modalities, Body-first PD was associated with a more symmetric and widespread pattern of neurodegeneration, while Brain-first PD demonstrated higher degrees of asymmetry. The two subtypes also exhibited diverging patterns of cerebral glucose metabolism, functional connectivity networks, and altered cortical α- and β-frequency networks. Body-first PD also showed decreased Global β-band EEG activity and a higher degree of colonic involvement. Interestingly, while both groups presented elevated plasma GFAP and NfL levels, the increase was more pronounced on the Body-first PD group. This subtype was also associated with lower mean right and left VN CSAs and a distinct microbiome gene biomarker profile. Both groups also differed in bile acid composition when compared to HCs, but the compositions appeared similar between the two subtypes. Finally, Body-first PD was associated with an increase in opportunistic pathogens—indicating intestinal dysbiosis—and a decrease in common commensal bacteria.

In Body-first PD, ASyn pathology has been speculated to propagate in a Braak-like, bottom-up trajectory, with initial involvement of the enteric nervous system. It then ascends via bilateral dorsal motor nuclei of the vagus nerve to brainstem structures such as raphe nuclei, locus coeruleus, and pedunculopontine nucleus. Importantly, these regions show signs of degeneration prior to the SNpr, the main driver of the classical PD motor manifestations. As those structures have been strongly linked to common PD NMS such as RBD, hallucinations, depression, anxiety, and apathy [43,44,45,46,47], the proposed sequence offers a coherent explanation for the clinical profile of early-stage Body-first PD, namely the higher prevalence of NMS but lower burden of motor symptoms. Under these circumstances, a longer prodromal stage can also be predicted in Body-first PD, which is consistent with the overall older age at PD diagnosis found in our review. Diffuse and bilateral propagation of ASyn pathology in Body-first PD could also account for the more rapid motor and non-motor symptom progression, as well as the increased risk of cognitive impairment. RBD, which is considered the principal differentiating factor between the two subtypes, has been repeatedly associated with multiple other NMS [48,49,50], suggesting a possible common dysfunctional neural network. Cognitive impairment has similarly been linked to common NMS in Body-first PD in previous literature [51,52,53]; however, the precise neural substrates of these associations remained mostly unexplored and warrant further investigation. LUTS were notably more prevalent in Body-first PD across studies. Peripheral autonomic dysfunction provides a plausible explanation, given the considerable evidence of autonomic involvement in this subtype. However, direct causal links remain speculative. Interestingly, LUTS appeared to exert a lesser impact on quality of life in early disease stages, as indicated by patient-reported outcome measures.

Imaging findings further support a more symmetric, widespread disease process in Body-first PD. Comparisons between individuals with iRBD—considered a prodromal stage of Body-first PD—and a mixed PD group revealed more symmetrical involvement in imaging as well as motor assessment scores in the former group. This observation aligns with the proposed bilateral ascending propagation pattern of ASyn pathology. Especially considering the LC, a region strongly implicated in RBD pathophysiology [46,47], a particularly pronounced and symmetrical involvement was observed in Body-first PD, accompanied by substantial alterations in functional connectivity between it and other core brain regions associated with multiple NMS [54]. Reduced network connectivity involving the LC has been associated with both NMS burden and cognitive impairment [55,56]. Notably, evidence suggests that Asyn propagation does not appear to follow a simplified connectome-driven pathway [57]. Despite prominent anatomical connectivity, regions such as the cerebellum—highly interconnected with the LC—remain relatively spared in early-stage PD. This suggests that additional factors beyond network strength influence the vulnerability to pathology. Assessment of cortical glucose metabolism using FDG-PET, a valuable imaging modality for PD differential diagnosis and disease progression monitoring [58], has suggested a distinct pattern of hypometabolism in Body-first PD, involving temporal, occipital and parietal areas. While these alterations possibly reflect a more diffuse cortical ASyn burden, their clinical manifestations remain to be elucidated. In contrast, GMV loss patterns did not consistently diverge between subtypes, highlighting heterogeneous results across different imaging modalities. Future multimodal imaging and post-mortem studies will be required to validate those findings. Neurophysiological studies have been recognized as valuable tools for assessing both motor symptom progression and cognitive decline in PD patients [59,60]. Specifically, quantitative EEG studies have demonstrated reduced global and site-specific β-band activity in Body-first PD, correlating with lower motor and cognitive assessment scores. Even though EEG slowing has been associated with cognitive impairment in PD [61], evidence specifically about β-band reductions is still scarce. Nonetheless, given the involvement of brainstem regions in β-band wave slowing in PD patients with RBD [62], it is safe to assume that these results are compatible with early brainstem involvement in the Body-first subtype. Alterations in specific cortical α- and β-band networks further differentiated the two groups. Notably, Body-first PD showed reduced connectivity values in the former and lower AIs in the latter network. Decreased α-band connectivity in Body-first PD may reflect early cholinergic system dysfunction, as it has been correlated with cholinergic system impairment in neurodegenerative diseases [63]. Correlations between α-band network connectivity and cognitive decline as well as UPDRS posture/gait scores in Body-first PD may be attributed to the well-established connection between the cholinergic system and cognition [64], and the early involvement of brainstem structures such as the PPN—a key regulator of gait and posture stability [65,66]—respectively.

An interesting conclusion emerges by comparing the PDRBDpre < 10 yrs and PDRBDpre ≥ 10 yrs groups, as the former demonstrates features that closely resemble the putative Body-first PD subtype, exhibiting greater putaminal involvement and a more symmetric pattern of denervation. Perhaps the PDRBDpre ≥ 10 yrs group represents a subset of PD patients in whom iRBD and Brain-first PD onset emerge as two temporally distinct, independent entities? This interpretation remains speculative and requires confirmation by future longitudinal studies.

The proposed involvement of the VN in early-stage PD and especially the Body-first subtype aligns with findings of reduced VN CSA in Body-first PD patients in our review, consistent with neurodegenerative processes affecting peripheral autonomic pathways [67,68]. Interestingly, vagal atrophy has been proposed as a potential differentiating biomarker between subtypes. Further validation in larger, longitudinal cohorts is required. Plasma biomarkers such as GFAP and NfL also demonstrated potential discriminatory value. Both markers were elevated in PD, with more pronounced increases observed in Body-first PD. GFAP reflects reactive astrocytosis [69,70], hinting at neuroinflammation playing a vital role in the pathogenesis of PD, specifically the Body-first subtype. NfL is indicative of neuronal damage and neurodegeneration, and it has been linked to faster motor progression and cognitive impairment in PD patients [71,72]. Although promising, the clinical utility of these biomarkers remains to be established in future studies. In our review, elevated microbiome gene biomarkers in Body-first PD were most commonly associated with curli fimbriae biosynthesis, lipopolysaccharide production, flagellar assembly and bacterial invasion pathways. All of the above contribute to chronic inflammation through TLR signaling [73], as well as ASyn aggregation and propagation. Chronic inflammation in the periphery has been described as being responsible for neurodegeneration through multiple pathways, including neuroinflammation and oxidative stress [74]. On the contrary, reductions were observed in gene biomarkers involved in putrescine, spermidine, and SCFA production. Putrescine and spermidine homeostasis is associated with numerous neuroprotective effects, such as anti-inflammatory responses, reactive oxygen species (ROS) removal, cell survival and autophagy [75]. SCFAs have a similar role, being involved in immune cell modulation, blood–brain barrier (BBB) strengthening, and mitochondria protection [76,77]. The bile acid composition differed between PD patients and HCs, with the two subtypes exhibiting diverging elevation patterns when compared to HCs. Commonly elevated bile acids included GCA and GCDCA, known for their involvement in gut inflammation [78,79], as well as GDCA, associated with BBB permeability modulation and cognitive decline [80]. However, when comparing the two subgroups, bile acid profiles were similar, suggesting a shared metabolic alteration pathway.

The “gut–brain axis” theory has proposed several ways of communication between the gut microbial community and the brain, hinting at possible mechanisms of neurodegeneration mediated by intestinal dysbiosis [81,82]. According to our data, even though the results concerning taxonomic and communal levels are mostly inconsistent, the two groups diverged significantly at the genus and species levels. Body-first PD demonstrated an abundance of genera and species associated with intestinal dysbiosis, inflammation and LPS production [83,84], alongside reductions in species implicated in gut barrier integrity, SCFA production and anti-inflammatory responses [76,85,86]. These findings support a link between the gut dysbiotic environment and the malignant clinical trajectory observed in this subtype, although further investigation is required for validation.

Several limitations should be acknowledged. First, the limited number of reviewers involved in study screening and data extraction introduces a potential risk of study selection bias. Second, a small number of studies—predominantly cross-sectional in design—was included in the review. Consequently, the strength of causal inference of our findings is unfortunately limited. Third, subtype classification relied primarily on the RBD status, determined using variable means and not always the gold-standard polysomnography. Furthermore, none of the included studies provided neuropathological verification of the proposed subtypes, thereby increasing the risk of misclassification. Fourth, although the focus of this review was de novo PD, inclusion criteria were expanded to encompass early PD with a disease duration up to six years, since study availability on de novo PD was limited. Hopefully, future reviews will include new cohorts and provide opportunities for explicit study of either de novo or early PD patients. Fifth, despite the use of universally accepted diagnostic criteria for PD across the included studies, distinguishing PD from other movement disorders, such as Progressive Supranuclear Palsy-Parkinsonism Predominant, may be challenging during early disease stages [87]. Therefore, the potential of diagnostic misclassification among included participants cannot be entirely ruled out. Sixth, the inclusion of gray literature was necessary to reduce publication bias, but may introduce additional methodological uncertainty, despite thorough risk-of-bias assessment. Seventh, no treatment-related studies or randomized-controlled trials (RCTs) were eligible for inclusion. This reflects the early stage of literature in this field and emphasizes the need for further research on the subject, as it could have immense implications for PD patients. Finally, selective outcome reporting within included studies cannot be excluded due to the small number of authors, potentially contributing to reporting bias. These limitations underscore the need for further longitudinal and interventional studies regarding this promising new classification theory, as it might imply new breakthroughs in the understanding and management of PD.

## 5. Conclusions

In conclusion, this systematic review highlights that the Brain-first vs. Body-first PD hypothesis, despite its recent emergence, provides new opportunities for conceptualizing clinical heterogeneity in PD. The two subtypes appear to differ in ASyn propagation trajectories, leading to distinct clinical phenotypes, symptom progression patterns and biological signatures. Emerging evidence suggests promising imaging, neurophysiological, biological, genetic and microbiome-related biomarkers that may facilitate subtype differentiation. Future studies will be essential to solidify the neuropathological basis of this theory and to elucidate whether these alterations translate into subtype-specific prognostic stratification and personalized management strategies. If validated, the Body-first/Brain-first PD framework has the potential to reshape both current knowledge and clinical practice on the subject of PD.

## Figures and Tables

**Table 1:** A summary of all extracted data from the included studies. Multiple values in age, sex distribution and disease duration were extracted due to the variable subtype differentiation methods. Disease duration was defined according to the original studies. PD = Parkinson’s Disease, RBD = REM Sleep Behavioral Disorder, PD-RBD+ = PD with RBD, PD-RBD- = PD without RBD, PD-RBDpre = PD with RBD preceding motor symptom onset, PD-RBDpost = PD with RBD after motor symptom onset, prRBD+ = PD with probable RBD, prRBD- = PD probably without RBD, PD-BrF = Brain-first PD, iRBD = Idiopathic RBD, HC = Healthy Controls, MD = Movement Disorders, MDS = Movement Disorders Society, RBDSQ = RBD Screening Questionnaire, RBD-1Q = RBD 1 Question screening tool, RBDQ-HK = RBD Questionnaire—Hong Kong, MIBG = Meta-Iodobenzylguanidine Scintigraphy, PSG = Polysomnography, FDOPA = Fluorodopa, PPMI = Parkinson’s Progression Markers Initiative. (-) = No data was provided. * Presented in mean (SD) or mean (range), according to the original studies. ^†^ Refers to the method(s) used for subtype classification. ^‡^ Included a cohort of pure PD patients as a comparator group to a cohort of iRBD individuals. ^§^ Included a separate, undetermined group characterized by divergent RBDSQ and MIBG scintigraphy findings.

Author (Year)	Country	Study Design	Population (Body-First PD/Brain-First PD/iRBD/HC)	PD Diagnosis Criteria	Subtypes Included	Age *	Sex Distribution (♂/♀)	Disease Duration in Years *	Exposure(s) Measured ^†^
Cicero et al. (2023) [20]	Italy	Cohort	115 (21/94/0/0)	UK Brain Bank	PD-RBD+	62.8 (9.4)	38/25	4.1 (4.2)	RBDSQ
					PD-RBDpre	66.7 (9.0)	13/8	2.7 (2.0)	
					PD-RBDpost	60.9 (9.2)	25/17	4.9 (4.8)	
					PD-RBD-	62.2 (10.2)	27/25	3.1 (3.5)	
Martinez-Nunez et al. (2025) [21]	USA	Cohort	452 (180/272/0/0)	(-)	Early prRBD+	(-)	(-)	≤2	RBDSQ
					Early prRBD-				
Xu et al. (2024) [22]	China	Cohort	137 (73/64/0/0)	UK Brain Bank and MDS	PD-RBD+	61.29 (10.89)	53/20	5.31 (5)	RBDSQ
					PD-RBD-	63.42 (6.68)	32/32	5.45 (4.18)	
Horsager et al. (2020) [23]	Germany	Cross-sectional	59 (13/24/22/0)	MDS	PD-RBD+	72.6 (5.3)	10/3	0.58 (0.16, 0.58)	PSG + RBDSQ
					PD-RBD-	62.3 (7.8)	18/6	0.20 (0.08, 0.70)	
					iRBD	68.6 (8.6)	18/4		
Sun et al. (2023) [24]	China	Cross-sectional	244 (58/64/53/69)	MDS	PD-RBD+	63.14 (7.2)	32/26	4 (0.16, 29.18)	RBDQ-HK + PSG
					PD-RBD-	63.33 (7.82)	30/34	3.82 (0.12, 21.16)	
					iRBD	65.49 (8.43)	32/21		
					HC	62.92 (6.89)	32/37		
Banwinkler et al. (2022) [25]	Germany	Cross-sectional	244 (67/67/0/110)	(-)	PD	63.18 (11.86)	162/97	0.34 (0.56)	RBDSQ
					HC	64.33 (11.09)	73/37		
Knudsen et al. (2021) [26]	Germany	Cross-sectional	FDOPA dataset 72 (11/22/21/18) PPMI dataset ^‡^ 637 (419 pure PD/25/193)	MDS	FDOPA dataset	FDOPA dataset	FDOPA dataset	FDOPA dataset	PSG + RBDSQ
					PD-RBD+	73 (5.4)	8/3	0.33 (0.12, 0.58)	
					PD-RBD-	63 (8.0)	16/6	0.16 (0.08, 0.58)	
					iRBD	70 (9)	17/4		
					HC	64 (6)	14/4		
					PPMI dataset	PPMI dataset	PPMI dataset	PPMI dataset	
					PD	61.1 (9.7)	268/151	(-)	
					iRBD	69 (4.5)	20/5		
					HC	60.2 (11.3)	123/70		
Woo et al. (2024) [27]	South Korea	Cross-sectional	150 (98/52/0/0)	MD specialist	PD-RBD+	67.05 (5.70)	56/42	1.06 (0.71)	RBD1Q
					PD-RBD-	66.50 (5.33)	24/28	1.17 (0.64)	
Conti et al. (2025) [28]	Italy	Cross-sectional	98 (28/35/0/35)	MDS	PD-RBD+	65 (58.3, 71.8)	20/8	1 (0.98, 2)	RBDSQ
					PD-RBD-	62 (47, 69)	22/13	0.5 (0.46, 1)	
					HC	60.0 (6.8)	21/14		
Terranova et al. (2024) [29]	Italy	Cross-sectional	83 (14/69/0/0)	UK Brain Bank	PD-RBDpre	65.6 (9.9)	8/6	2 (2)	RBDSQ
					PD-RBDpost	60.1 (9.3)	18/13	5 (5.2)	
					PD-RBD-	62.1 (9.3)	20/18	3 (2.7)	
Cicero et al. (2024) [30]	Italy	Cross-sectional	56 (10/46/0/0)	UK Brain Bank	PD-RBDpre	67.0 (6.6)	8/2	2.7 (2.2)	RBDSQ
					PD-RBDpost	59.5 (9.7)	11/8	4.2 (4.7)	
					PD-RBD-	62.5 (8.8)	13/14	1.7 (1.3)	
					(PD-BrF)	61.3 (9.2)	24/22	2.7 (3.5)	
Park et al. (2024) [31]	South Korea	Cross-sectional	36 (15/9/0/0 + 12 Undetermined ^§^)	UK Brain Bank	Body-first PD	67.00 (65.50, 70.50)	(-)	1.63 (1.26, 2.56)	RBDSQ + MIBG
					Brain-first PD	58.00 (50.00, 61.00)		1.85 (0.41, 2.12)	
					Undetermined	63.50 (55.50, 68.75)		1.63 (0.49, 2.46)	
Li et al. (2025) [32]	China	Cross-sectional	116 (43/58/0/15)	UK Brain Bank	PD-RBD+	60.72 (10.25)	27/16	1.08 (0.79, 2.04)	RBDSQ
					PD-RBD-	59.81 (7.93)	31/27	1.16 (0.75, 1.83)	
					HC	62.05 (8.52)	6/9		
Dong et al. (2024) [33]	China	Cross-sectional	171 (27/48/0/96)	MDS	PD	66.93 (7.27)	38/37		RBDSQ
					PD-RBDpre	68.67 (5.97)	17/10	2.00 (1.00, 3.00)	
					PD-RBDpost	64.20 (7.71)	4/6	1.50 (0.88, 3.25)	
					PD-RBD-	66.42 (7.86)	17/21	2.00 (0.50, 3.00)	
					HC	65.48 (10.39)	42/54		
Kim et al. (2024) [34]	South Korea	Cross-sectional	55 (37/18/0/0)	UK Brain Bank	Body-first PD	69.8 (7.7)	15/22	1.3 (1.2)	RBD-1Q + MIBG
					Brain-first PD	66.7 (10.9)	6/12	1.1 (1.1)	
Duan et al. (2023) [35]	China	Cross-sectional	67 (18/21/0/28)	MDS	PD-RBD+	59.56 (10.98)	8/10	3.00 (5.00)	PSG
					PD-RBD-	61.29 (10.34)	12/9	2.00 (2.00)	
					HC	60.18 (7.95)	15/13		

## Data Availability

The original contributions presented in this study are included in the article/Supplementary Material. Further inquiries can be directed to the corresponding author(s).

## Supplementary Materials

The following supporting information can be downloaded at https://www.mdpi.com/article/10.3390/medicina62061116/s1, Supplementary Figure S1: Detailed risk-of-bias assessment for all studies; Supplementary Table S1: Search strategy; Supplementary Table S2: Risk-of-bias assessment summary; Supplementary Table S3: GRADE interactive summary of findings—certainty of evidence; Supplementary PRISMA 2020 Checklist.
