# Supplementary material for: “Brain-First” vs. “Body-First” PD: Definitions and Implications in Everyday Clinical Practice: A Systematic Review
Source: Medicina (Kaunas). 2026 Jun 8;62(6):1116. doi: 10.3390/medicina62061116 (PMC13303078; doi:10.3390/medicina62061116)
Supplement: Supplementary file 1 [file medicina-62-01116-s001.zip › medicina-4325147-supplementary(5).pdf]

Supplementary Table S1. Search Strategy

Search strategy: (PD OR Parkinson) AND ("Brain first" OR "Body first" OR "Brain-first" OR "Body-first" OR "Gut first" OR "Gut-first") with slight alterations for different databases.

Database	ID	Query	Results
**PUBMED**	#1	Parkinson	63416
	#2	PD	1425618
	#3	#1 OR #2	1452646
	#4	“Brain first” OR “Brain-first”	73
	#5	“Body first” OR “Body-first”	84
	#6	“Gut first” OR “Gut-first”	14
	#7	#4 OR #5 OR #6	126
	#8	#3 AND #7	90
**COCHRANE LIBRARY**	#1	Parkinson	13518
	#2	PD	43372
	#3	#1 OR #2	50555
	#4	“Brain first” OR “Brain-first”	9
	#5	“Body first” OR “Body-first”	16
	#6	“Gut first” OR “Gut-first”	0
	#7	#4 OR #5 OR #6	25
	#8	#3 AND #7	1
**DOAJ**	#1	Parkinson	3268
	#2	PD	30064
	#3	“Brain first” OR “Brain-first”	36
	#4	“Body first” OR “Body-first”	30
	#5	“Gut first” OR “Gut-first”	4
	#6	#1 AND #3	1
	#7	#1 AND #4	1
	#8	#1 AND #5	1
	#9	#2 AND #3	14
	#10	#2 AND #4	16
	#11	#2 AND #5	3
	#12	#6+#7+#8+#9+#10+#11	36
**GOOGLE SCHOLAR**	#1	Parkinson	375000
	#2	PD	1330000
	#3	#1 OR #2	1640000
	#4	“Brain first” OR “Brain-first”	4310
	#5	“Body first” OR “Body-first”	9640
	#6	“Gut first” OR “Gut-first”	3750
	#7	#4 OR #5 OR #6	14500
	#8	#3 AND #7	3510
	Most relevant results		1000

Supplementary Table S2. Risk of Bias Assessment Summary

Study ID	Study Design	Checklist Used	Criterion 1	Criterion 2	Criterion 3	Criterion 4	Criterion 5	Criterion 6	Criterion 7	Criterion 8	Criterion 9	Criterion 10	Criterion 11	Score
Cicero et al. (2023)[20]	Cohort	JBI Cohort Checklist	YES	YES	NO	YES	YES	N/A	YES	YES	NO	NO	YES	**7/11**
Martinez-Nunez et al. (2025)[21]	Cohort	JBI Cohort Checklist	YES	YES	NO	YES	NO	N/A	YES	YES	YES	N/A	YES	**7/11**
Xu et al. (2024)[22]	Cohort	JBI Cohort Checklist	YES	YES	NO	YES	YES	N/A	YES	YES	YES	N/A	YES	**8/11**

Study ID	Study Design	Checklist Used	Criterion 1	Criterion 2	Criterion 3	Criterion 4	Criterion 5	Criterion 6	Criterion 7	Criterion 8	Score
Horsager et al. (2020)[23]	Cross-sectional	JBI Cross-Sectional Checklist	YES	YES	YES	YES	YES	YES	YES	YES	**8/8**
Sun et al. (2023)[24]	Cross-sectional	JBI Cross-Sectional Checklist	YES	YES	YES	YES	YES	YES	YES	YES	**8/8**
Banwinkler et al. (2022)[25]	Cross-sectional	JBI Cross-Sectional Checklist	YES	YES	NO	YES	YES	YES	YES	YES	**7/8**
Knudsen et al. (2021)[26]	Cross-sectional	JBI Cross-Sectional Checklist	YES	YES	YES	YES	YES	YES	YES	YES	**8/8**

Study ID	Study Design	Checklist Used	Criterion 1	Criterion 2	Criterion 3	Criterion 4	Criterion 5	Criterion 6	Criterion 7	Criterion 8	Score
Woo et al. (2024) [27]	Cross-sectional	JBI Cross-Sectional Checklist	YES	YES	NO	YES	YES	NO	YES	YES	**6/8**
Conti et al. (2025) [28]	Cross-sectional	JBI Cross-Sectional Checklist	YES	YES	NO	YES	YES	YES	YES	YES	**7/8**
Terranova et al. (2024) [29]	Cross-sectional	JBI Cross-Sectional Checklist	YES	YES	NO	YES	YES	YES	YES	YES	**7/8**
Cicero et al. (2024) [30]	Cross-sectional	JBI Cross-Sectional Checklist	YES	YES	NO	YES	YES	YES	YES	YES	**7/8**
Park et al. (2024) [31]	Cross-sectional	JBI Cross-Sectional Checklist	YES	YES	YES	YES	YES	YES	YES	YES	**8/8**
Li et al. (2025) [32]	Cross-sectional	JBI Cross-Sectional Checklist	YES	YES	NO	YES	YES	YES	YES	YES	**7/8**
Dong et al. (2024) [33]	Cross-sectional	JBI Cross-Sectional Checklist	YES	YES	NO	YES	YES	YES	YES	YES	**7/8**

Study ID	Study Design	Checklist Used	Criterion 1	Criterion 2	Criterion 3	Criterion 4	Criterion 5	Criterion 6	Criterion 7	Criterion 8	Score
Kim et al. (2024) [34]	Cross-sectional	JBI Cross-Sectional Checklist	YES	YES	YES	YES	YES	YES	YES	YES	**8/8**
Duan et al (2023) [35]	Cross-sectional	JBI Cross-Sectional Checklist	YES	YES	YES	YES	YES	YES	YES	YES	**8/8**

A detailed summary of the Risk of Bias assessment of included studies, utilizing the JBI Critical Appraisal Checklists for Cohort and Analytical Cross-Sectional Studies. Each criterion was scored as 1 (YES) or 0 (NO or N/A). Cutoff values for inclusion were predefined at protocol as 7/11 for Cohort and 5/8 for Cross-sectional studies, respectively.

Supplementary Figure S1. Detailed Risk of Bias assessment for all studies

JBI Critical Appraisal Checklist for cohort studies

Author________Cicero et al.______________________Year___2023___ Record Number___#20____

	Yes	No	Unclear	Not applicable
1. Were the two groups similar and recruited from the same population?	☑	☐	☐	☐
2. Were the exposures measured similarly to assign people to both exposed and unexposed groups?	☑	☐	☐	☐
3. Was the exposure measured in a valid and reliable way?	☐	☑	☐	☐
4. Were confounding factors identified?	☑	☐	☐	☐
5. Were strategies to deal with confounding factors stated?	☑	☐	☐	☐
6. Were the groups/participants free of the outcome at the start of the study (or at the moment of exposure)?	☐	☐	☐	☑
7. Were the outcomes measured in a valid and reliable way?	☑	☐	☐	☐
8. Was the follow up time reported and sufficient to be long enough for outcomes to occur?	☑	☐	☐	☐
9. Was follow up complete, and if not, were the reasons to loss to follow up described and explored?	☐	☑	☐	☐
10. Were strategies to address incomplete follow up utilized?	☐	☑	☐	☐
11. Was appropriate statistical analysis used?	☑	☐	☐	☐

Overall appraisal: Include ☑ Exclude ☐ Seek further info ☐

JBI Critical Appraisal Checklist for cohort studies

Author____Martinez-Nunez et al.__________________Year__2025___ Record Number___#21____

	Yes	No	Unclear	Not applicable
1. Were the two groups similar and recruited from the same population?	☑	☐	☐	☐
2. Were the exposures measured similarly to assign people to both exposed and unexposed groups?	☑	☐	☐	☐
3. Was the exposure measured in a valid and reliable way?	☐	☑	☐	☐
4. Were confounding factors identified?	☑	☐	☐	☐
5. Were strategies to deal with confounding factors stated?	☐	☑	☐	☐
6. Were the groups/participants free of the outcome at the start of the study (or at the moment of exposure)?	☐	☐	☐	☑
7. Were the outcomes measured in a valid and reliable way?	☑	☐	☐	☐
8. Was the follow up time reported and sufficient to be long enough for outcomes to occur?	☑	☐	☐	☐
9. Was follow up complete, and if not, were the reasons to loss to follow up described and explored?	☑	☐	☐	☐
10. Were strategies to address incomplete follow up utilized?	☐	☐	☐	☑
11. Was appropriate statistical analysis used?	☑	☐	☐	☐

Overall appraisal: Include ☑ Exclude ☐ Seek further info ☐

JBI Critical Appraisal Checklist for cohort studies

Author____________Xu et al.______________________ Year__2024____ Record Number__#22_____

	Yes	No	Unclear	Not applicable
1. Were the two groups similar and recruited from the same population?	☑	☐	☐	☐
2. Were the exposures measured similarly to assign people to both exposed and unexposed groups?	☑	☐	☐	☐
3. Was the exposure measured in a valid and reliable way?	☐	☑	☐	☐
4. Were confounding factors identified?	☑	☐	☐	☐
5. Were strategies to deal with confounding factors stated?	☑	☐	☐	☐
6. Were the groups/participants free of the outcome at the start of the study (or at the moment of exposure)?	☐	☐	☐	☑
7. Were the outcomes measured in a valid and reliable way?	☑	☐	☐	☐
8. Was the follow up time reported and sufficient to be long enough for outcomes to occur?	☑	☐	☐	☐
9. Was follow up complete, and if not, were the reasons to loss to follow up described and explored?	☑	☐	☐	☐
10. Were strategies to address incomplete follow up utilized?	☐	☐	☐	☑
11. Was appropriate statistical analysis used?	☑	☐	☐	☐

Overall appraisal: Include ☑ Exclude ☐ Seek further info ☐

JBI Critical Appraisal Checklist for analytical cross sectional studies

Author_________Horsager et al.___________________Year__2020____ Record Number__#23____

	Yes	No	Unclear	Not applicable
1. Were the criteria for inclusion in the sample clearly defined?	☑	☐	☐	☐
2. Were the study subjects and the setting described in detail?	☑	☐	☐	☐
3. Was the exposure measured in a valid and reliable way?	☑	☐	☐	☐
4. Were objective, standard criteria used for measurement of the condition?	☑	☐	☐	☐
5. Were confounding factors identified?	☑	☐	☐	☐
6. Were strategies to deal with confounding factors stated?	☑	☐	☐	☐
7. Were the outcomes measured in a valid and reliable way?	☑	☐	☐	☐
8. Was appropriate statistical analysis used?	☑	☐	☐	☐

Overall appraisal: Include ☑ Exclude ☐ Seek further info ☐

JBI Critical Appraisal Checklist for analytical cross sectional studies

Author_________________Sun et al._______________ Year___2023___ Record Number___#24____

	Yes	No	Unclear	Not applicable
1. Were the criteria for inclusion in the sample clearly defined?	☑	☐	☐	☐
2. Were the study subjects and the setting described in detail?	☑	☐	☐	☐
3. Was the exposure measured in a valid and reliable way?	☑	☐	☐	☐
4. Were objective, standard criteria used for measurement of the condition?	☑	☐	☐	☐
5. Were confounding factors identified?	☑	☐	☐	☐
6. Were strategies to deal with confounding factors stated?	☑	☐	☐	☐
7. Were the outcomes measured in a valid and reliable way?	☑	☐	☐	☐
8. Was appropriate statistical analysis used?	☑	☐	☐	☐

Overall appraisal: Include ☑ Exclude ☐ Seek further info ☐

JBI Critical Appraisal Checklist for analytical cross sectional studies

Author____________Banwinkler et al.______________Year___2022___ Record Number__#25_____

	Yes	No	Unclear	Not applicable
1. Were the criteria for inclusion in the sample clearly defined?	☑	☐	☐	☐
2. Were the study subjects and the setting described in detail?	☑	☐	☐	☐
3. Was the exposure measured in a valid and reliable way?	☐	☑	☐	☐
4. Were objective, standard criteria used for measurement of the condition?	☑	☐	☐	☐
5. Were confounding factors identified?	☑	☐	☐	☐
6. Were strategies to deal with confounding factors stated?	☑	☐	☐	☐
7. Were the outcomes measured in a valid and reliable way?	☑	☐	☐	☐
8. Was appropriate statistical analysis used?	☑	☐	☐	☐

Overall appraisal: Include ☑ Exclude ☐ Seek further info ☐ ______________

JBI Critical Appraisal Checklist for analytical cross sectional studies

Author___________Knudsen et al.___________________Year__2021___ Record Number__#26____

	Yes	No	Unclear	Not applicable
1. Were the criteria for inclusion in the sample clearly defined?	☑	☐	☐	☐
2. Were the study subjects and the setting described in detail?	☑	☐	☐	☐
3. Was the exposure measured in a valid and reliable way?	☑	☐	☐	☐
4. Were objective, standard criteria used for measurement of the condition?	☑	☐	☐	☐
5. Were confounding factors identified?	☑	☐	☐	☐
6. Were strategies to deal with confounding factors stated?	☑	☐	☐	☐
7. Were the outcomes measured in a valid and reliable way?	☑	☐	☐	☐
8. Was appropriate statistical analysis used?	☑	☐	☐	☐

Overall appraisal: Include ☑ Exclude ☐ Seek further info ☐

JBI Critical Appraisal Checklist for analytical cross sectional studies

Author_________Woo et al. _________________________Year___2024___ Record Number__#27_

	Yes	No	Unclear	Not applicable
1. Were the criteria for inclusion in the sample clearly defined?	☑	☐	☐	☐
2. Were the study subjects and the setting described in detail?	☑	☐	☐	☐
3. Was the exposure measured in a valid and reliable way?	☐	☑	☐	☐
4. Were objective, standard criteria used for measurement of the condition?	☑	☐	☐	☐
5. Were confounding factors identified?	☑	☐	☐	☐
6. Were strategies to deal with confounding factors stated?	☐	☑	☐	☐
7. Were the outcomes measured in a valid and reliable way?	☑	☐	☐	☐
8. Was appropriate statistical analysis used?	☑	☐	☐	☐

Overall appraisal: Include ☑ Exclude ☐ Seek further info ☐

JBI Critical Appraisal Checklist for analytical cross sectional studies

Author_________Conti et al.________________________Year__2025___ Record Number___#28___

	Yes	No	Unclear	Not applicable
1. Were the criteria for inclusion in the sample clearly defined?	☑	☐	☐	☐
2. Were the study subjects and the setting described in detail?	☑	☐	☐	☐
3. Was the exposure measured in a valid and reliable way?	☐	☑	☐	☐
4. Were objective, standard criteria used for measurement of the condition?	☑	☐	☐	☐
5. Were confounding factors identified?	☑	☐	☐	☐
6. Were strategies to deal with confounding factors stated?	☑	☐	☐	☐
7. Were the outcomes measured in a valid and reliable way?	☑	☐	☐	☐
8. Was appropriate statistical analysis used?	☑	☐	☐	☐

Overall appraisal: Include ☑ Exclude ☐ Seek further info ☐

JBI Critical Appraisal Checklist for analytical cross sectional studies

Author________Terranova et al._____________________Year__2024___ Record Number__#29____

	Yes	No	Unclear	Not applicable
1. Were the criteria for inclusion in the sample clearly defined?	☑	☐	☐	☐
2. Were the study subjects and the setting described in detail?	☑	☐	☐	☐
3. Was the exposure measured in a valid and reliable way?	☐	☑	☐	☐
4. Were objective, standard criteria used for measurement of the condition?	☑	☐	☐	☐
5. Were confounding factors identified?	☑	☐	☐	☐
6. Were strategies to deal with confounding factors stated?	☑	☐	☐	☐
7. Were the outcomes measured in a valid and reliable way?	☑	☐	☐	☐
8. Was appropriate statistical analysis used?	☑	☐	☐	☐

Overall appraisal: Include ☑ Exclude ☐ Seek further info ☐

JBI Critical Appraisal Checklist for analytical cross sectional studies

Author________Cicero et al.________________________Year__2024___ Record Number___#30___

	Yes	No	Unclear	Not applicable
1. Were the criteria for inclusion in the sample clearly defined?	☑	☐	☐	☐
2. Were the study subjects and the setting described in detail?	☑	☐	☐	☐
3. Was the exposure measured in a valid and reliable way?	☐	☑	☐	☐
4. Were objective, standard criteria used for measurement of the condition?	☑	☐	☐	☐
5. Were confounding factors identified?	☑	☐	☐	☐
6. Were strategies to deal with confounding factors stated?	☑	☐	☐	☐
7. Were the outcomes measured in a valid and reliable way?	☑	☐	☐	☐
8. Was appropriate statistical analysis used?	☑	☐	☐	☐

Overall appraisal: Include ☑ Exclude ☐ Seek further info ☐

JBI Critical Appraisal Checklist for analytical cross sectional studies

Author__________Park et al.______________________Year__2024___ Record Number___#31___

	Yes	No	Unclear	Not applicable
1. Were the criteria for inclusion in the sample clearly defined?	☑	☐	☐	☐
2. Were the study subjects and the setting described in detail?	☑	☐	☐	☐
3. Was the exposure measured in a valid and reliable way?	☑	☐	☐	☐
4. Were objective, standard criteria used for measurement of the condition?	☑	☐	☐	☐
5. Were confounding factors identified?	☑	☐	☐	☐
6. Were strategies to deal with confounding factors stated?	☑	☐	☐	☐
7. Were the outcomes measured in a valid and reliable way?	☑	☐	☐	☐
8. Was appropriate statistical analysis used?	☑	☐	☐	☐

Overall appraisal: Include ☑ Exclude ☐ Seek further info ☐

JBI Critical Appraisal Checklist for analytical cross sectional studies

Author_________Li et al.__________________________Year__2025___ Record Number___#32___

	Yes	No	Unclear	Not applicable
1. Were the criteria for inclusion in the sample clearly defined?	☑	☐	☐	☐
2. Were the study subjects and the setting described in detail?	☑	☐	☐	☐
3. Was the exposure measured in a valid and reliable way?	☐	☑	☐	☐
4. Were objective, standard criteria used for measurement of the condition?	☑	☐	☐	☐
5. Were confounding factors identified?	☑	☐	☐	☐
6. Were strategies to deal with confounding factors stated?	☑	☐	☐	☐
7. Were the outcomes measured in a valid and reliable way?	☑	☐	☐	☐
8. Was appropriate statistical analysis used?	☑	☐	☐	☐

Overall appraisal: Include ☑ Exclude ☐ Seek further info ☐

JBI Critical Appraisal Checklist for analytical cross sectional studies

Author________Dong et al. _______________________Year__2024___ Record Number___#33___

	Yes	No	Unclear	Not applicable
1. Were the criteria for inclusion in the sample clearly defined?	☑	☐	☐	☐
2. Were the study subjects and the setting described in detail?	☑	☐	☐	☐
3. Was the exposure measured in a valid and reliable way?	☐	☑	☐	☐
4. Were objective, standard criteria used for measurement of the condition?	☑	☐	☐	☐
5. Were confounding factors identified?	☑	☐	☐	☐
6. Were strategies to deal with confounding factors stated?	☑	☐	☐	☐
7. Were the outcomes measured in a valid and reliable way?	☑	☐	☐	☐

	Yes	No	Unclear	Not applicable
8. Was appropriate statistical analysis used?	☑	☐	☐	☐

Overall appraisal: Include ☑ Exclude ☐ Seek further info ☐

JBI Critical Appraisal Checklist for analytical cross sectional studies

Author___________Kim et al.________________________Year__2024___ Record Number___#34___

	Yes	No	Unclear	Not applicable
1. Were the criteria for inclusion in the sample clearly defined?	☑	☐	☐	☐
2. Were the study subjects and the setting described in detail?	☑	☐	☐	☐
3. Was the exposure measured in a valid and reliable way?	☑	☐	☐	☐
4. Were objective, standard criteria used for measurement of the condition?	☑	☐	☐	☐
5. Were confounding factors identified?	☑	☐	☐	☐
6. Were strategies to deal with confounding factors stated?	☑	☐	☐	☐

	Yes	No	Unclear	Not applicable
7. Were the outcomes measured in a valid and reliable way?	☑	☐	☐	☐
8. Was appropriate statistical analysis used?	☑	☐	☐	☐

Overall appraisal: Include ☑ Exclude ☐ Seek further info ☐

JBI Critical Appraisal Checklist for analytical cross sectional studies

Author_______Duan et al______________ ___________Year__2023___ Record Number___#35___

	Yes	No	Unclear	Not applicable
1. Were the criteria for inclusion in the sample clearly defined?	☑	☐	☐	☐
2. Were the study subjects and the setting described in detail?	☑	☐	☐	☐
3. Was the exposure measured in a valid and reliable way?	☑	☐	☐	☐
4. Were objective, standard criteria used for measurement of the condition?	☑	☐	☐	☐
5. Were confounding factors identified?	☑	☐	☐	☐

6. Were strategies to deal with confounding factors stated?	☑	☐	☐	☐
7. Were the outcomes measured in a valid and reliable way?	☑	☐	☐	☐
8. Was appropriate statistical analysis used?	☑	☐	☐	☐

Overall appraisal: Include ☑ Exclude ☐ Seek further info ☐

JBI Critical Appraisal Checklist for analytical cross sectional studies

Author_______Lee et al.________________________Year___2024___ Record Number___#42___

	Yes	No	Unclear	Not applicable
1. Were the criteria for inclusion in the sample clearly defined?	☐	☑	☐	☐
2. Were the study subjects and the setting described in detail?	☑	☐	☐	☐
3. Was the exposure measured in a valid and reliable way?	☐	☑	☐	☐
4. Were objective, standard criteria used for measurement of the condition?	☑	☐	☐	☐

5.	Were confounding factors identified?	☐	☑	☐	☐
6.	Were strategies to deal with confounding factors stated?	☐	☑	☐	☐
7.	Were the outcomes measured in a valid and reliable way?	☑	☐	☐	☐
8.	Was appropriate statistical analysis used?	☑	☐	☐	☐

Overall appraisal: Include ☐ Exclude ☑ Seek further info ☐

Supplementary Table S3. GRADE interactive Summary of Findings - Certainty of Evidence

Outcomes	Plain language statements	Effect	Certainty of the evidence GRADE
**Differences in clinical characteristics and disease progression**[*]<br><br>Follow-up: Includes cross sectional and longitudinal data, up to 5 years	Evidence suggests that Body-first PD tends to be associated with a higher burden of non-motor symptoms and a higher frequency of cognitive impairment over time. Some studies also report faster progression of motor symptoms, while others show no significant differences in motor severity or disease stage between subtypes. Findings regarding demographic characteristics are inconsistent across studies. Overall, results are heterogenous, with several studies reporting no statistically significant differences in clinical measures between Body-first and Brain-first PD.<br><br>*Evidence is based on observational studies with heterogenous methods and variable sample sizes.*	**Moderate / High impact**<br><br>Several studies reported significant differences between groups in non-motor symptom severity as well as cognitive manifestations and motor symptom severity progression.	⨁⨁◯◯<br>**Low**<br><br>Downgraded due to observational study designs, inconsistency of results between studies and imprecision.
**Imaging differences**[†]<br><br>Follow-up: Not applicable, includes only cross - sectional data	Imaging findings suggest that Body-first PD is strongly associated with more symmetric and widespread neurodegenerative pathology, broader autonomic and colonic involvement, altered LC functional connectivity patterns and lower α- and β-band activity values when compared to Brain-first PD. However, results were heterogenous and not fully consistent between studies.<br><br>*Imaging findings are derived from small-to-moderate sized observational studies using diverse imaging modalities, limiting cross-study comparability.*	**Uncertain, probably low**<br><br>Although multiple imaging modalities suggested differences between the two groups, findings were heterogenous and do not translate into clinical decision-making or routine subtyping.	⨁⨁◯◯<br>**Low**<br><br>Downgraded due to study design, inconsistency of results, variability across imaging methods, imprecision and indirectness of outcomes.

Outcomes	Plain language statements	Effect	Certainty of the evidence GRADE
**Biomarker differences**‡<br><br>Follow-up: Not applicable, includes only cross - sectional data	Evidence suggests that Body-first PD is associated with increased levels of plasma GFAP and NfL, greater vagus nerve involvement, as well as alterations in microbiome gene biomarkers leading to increased inflammatory responses and a-synuclein aggregation and decreased SCFA production when compared with Brain-first PD. However, not all biomarker groups assessed were significantly different between the two subtypes (i.e comparable bile acid profiles).<br><br>*Results were derived from a small number of observational studies, focusing on different types of biomarkers. Some showed significant differences between subtypes, while others did not.*	**Moderate**<br><br>Elevated GFAP and NfL levels, reduced vagus nerve CSA, and altered microbiome gene pathways in Body-first PD suggest a distinct pathophysiological mechanism with potential diagnostic use, but are not yet validated.	⨁⨁○○<br>**Low**<br><br>Downgraded due to indirectness, imprecision, observational study design, and inconsistency.
**Microbiome differences**§<br><br>Follow-up: Not applicable, includes only cross - sectional data	Body-first PD tends to associate with a distinct gut microbiome composition in the genus and species level when compared with Brain-first PD, showing an increase in bacteria related to gut inflammation and promotion of a-synuclein aggregation, and a decrease in commensal bacteria responsible for production of beneficial metabolites. Nonetheless, comparisons regarding taxonomic and communal levels provided mixed results.<br><br>*Some studies found significant differences between the Body-first and Brain-first PD groups in overall microbiome composition as well as specific bacterial groups, while other studies found no difference in diversity indexes. The findings are based on a small number of studies with high variability in results.*	**Low to moderate**<br><br>The alterations observed in gut microbiome composition in Body-first PD patients are linked in the literature to gut dysfunction and a-synuclein aggregation, hinting to a distinct pathophysiological mechanism, but have not yet been approved as diagnostic markers.	⨁⨁○○<br>**Low**<br><br>Downgraded due to imprecision, inconsistency between studies, indirectness, as well as study design.

Certainty of evidence assessment using the GRADE framework, evaluating risk of bias, inconsistency, indirectness, imprecision, and publication bias. The interactive Summary of Findings (iSoF) online tool was utilized in the creation of this figure. PD = Parkinson's Disease, LC = Locus Coeruleus, SCFA = Short Chain Fatty Acids, CSA = Cross-Sectional Area

[*] *Includes demographic, motor symptom, non-motor symptom, cognitive decline, as well as disease progression differences.*

[†] *Includes differences in colonic transit times, FP-CIT PET, MIBG scintigraphy and 18F-FDG PET findings, symmetry in dopaminergic dysfunction, functional connectivity as well as neurophysiological studies.*

[‡] *Includes plasma biomarkers, vagus nerve CSA, bile acids, and microbiome gene biomarkers.*

[§] *Includes differences in microbiome regarding α-diversity and β-diversity, as well as regarding genera and species.*
